# Detection of very delayed coronary stent fracture using a novel fluoroscopic stent visualization technique against intravascular ultrasound: A case series

**DOI:** 10.1097/MD.0000000000006804

**Published:** 2017-05-26

**Authors:** Feifei Yang, Liwei Zhang, Dangsheng Huang, Dong Shen, Yongjiang Ma, Minjun Xiong, Chunhong Zhang, Miao Tian

**Affiliations:** Department of Cardiology, First Affiliated Hospital of Chinese PLA General Hospital, Beijing, China.

**Keywords:** case report, intravascular ultrasound, percutaneous coronary intervention, stent fracture, stentboost

## Abstract

**Rationale:**

Stent fracture has received increased concern as it may be an important risk factor for late stent failure, intravascular ultrasound (IVUS) is always recommended to confirm the diagnosis of stent fracture. StentBoost can detect stent fractures more easily due to the enhanced stent strut visibility, compared with coronary angiography (CAG). Few cases were reported to compare the advantages of StentBoost vis-à-vis IVUS in detecting stent fracture.

**Patient Concerns:**

We reported 3 cases that were confirmed the diagnosis of stent fracture by StentBoost, which were preliminarily suspected by angiography, including one case that lacked the IVUS evidence of stent fracture.

**Diagnoses:**

3 cases of stent fracture presented with asymptomatic, angina and acute myocardial infarction, respectively.

**Intervention:**

Stents were implanted in the patients of case 2 and case 3, but the patient of case 1 was not given any intervention.

**Outcomes:**

No recurrent angina or myocardial infarction during outpatient follow-up.

**Lessons:**

StentBoost may distinguish partial, complete, or multiple stent fracture, even which sometimes is not obvious in IVUS, StentBoost is a useful and handy tool for identifying the stent struts.

## 1 Introduction

Stent fracture has been a new focus of concern due to its contributing factor in the late stent failure, although the incidence is low.^[1,2]^ It is difficult to detect stent fractures using a conventional x-ray fluoroscopy because of its poor stent radiopacity. Intravascular ultrasound (IVUS) or optical coherence tomography (OCT) is always recommended to confirm the diagnosis of stent fracture that was suspected by angiography.^[3]^ But IVUS or OCT is limited by professional and technical expertise, cost, and procedural time. StentBoost (SB, Philips Medical Systems, Eindoven, The Netherlands), an improved angiographic visualization technique, can make up for these deficiencies. StentBoost does not require any additional expensive hardware. It can create a high-quality image of deployed stents by superimposing motion-corrected acquisition frames; thus, it can easily detect the stent fracture due to the enhanced angiographic visualization of the stent struts.

This study presents 3 case reports that were confirmed by the diagnosis of stent fracture by StentBoost, which were preliminarily suspected by angiography, including 1 case that lacked the IVUS evidence of stent fracture. This study discusses the benefits of using the StentBoost in such situations. We also obtained written informed consent from those patients for publication of this case report and any accompanying images.

## 2 Case report

### 2.1 Case 1—enhanced visualization of stent fracture with no symptoms

**Figure 1:**
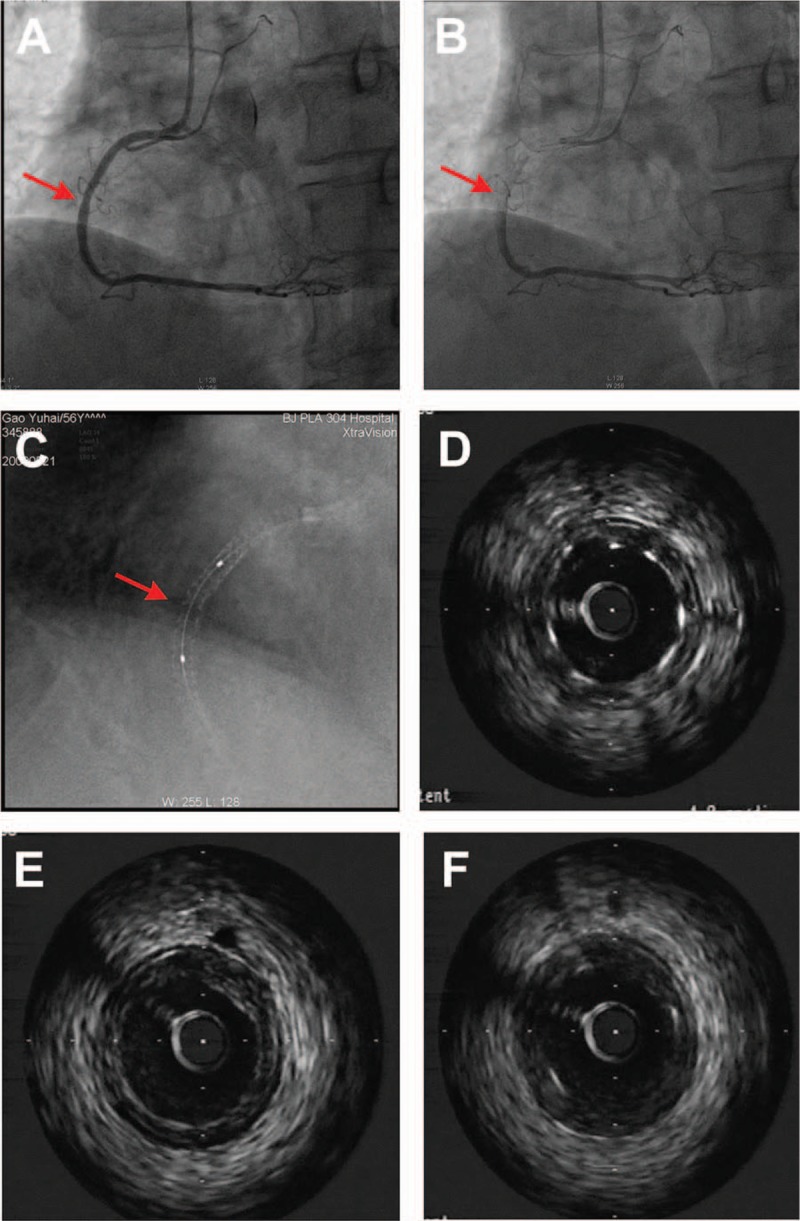
A, B, Coronary angiography showed 30% mild restenosis in the old stent of RCA (red arrow). C, StentBoost showed stent fracture in the place of mild restenosis in RCA (red arrow). D–F, IVUS images revealed mild neointimal hyperplasia in stents and the local absence of metallic stent struts (E). IVUS = intravascular ultrasound, RCA = right coronary artery.

A 57-year-old man, a cigarette smoker, with hypertension presented with chest pain after mild effort; in September 2007, he had primary percutaneous coronary intervention (PCI) to the mid-portion of right coronary artery (RCA) with 2 overlapping firebird stents (Shanghai MicroPort Medical, Shanghai, China) because of 99% stenosis. After 2 years, he was admitted just for aroutine angiographic follow-up without any discomfort. In May 2009, the coronary angiography showed mild restenosis in the first stent in RCA (Fig. 1A and B). In this study, StentBoost was used on PHILIPH Allura Xper FD20 system to enhance stent visualization; a stent fracture was found in the place of mild restenosis in RCA (Fig. 1C, red arrow). Then IVUS (iLab, Boston Scientific Scimed Inc, Fremont, CA) was performed to confirm the results of StentBoost. IVUS revealed mild neointimal hyperplasia in the stents (<50%) and the local absence of metallic stent struts (Fig. 1D–F); Fig. 1E shows the position of the stent fracture.

Because the patient did not feel any discomfort in general activities and the restenosis was mild, he continued with his prescription of aspirin, atorvastatin, and benazepril, and was advised to follow up periodically. During the 6 years of follow-up, he had no recurrent angina and myocardial infarction.

### 2.2 Case 2—multiple stent fracture caused angina due to stent restenosis

**Figure 2:**
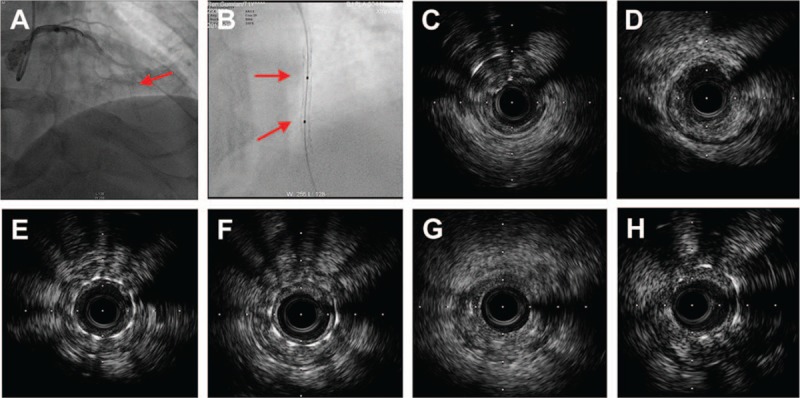
A, The angiogram showed that the old stent in the distal LAD artery was discontinuous (red arrow). B, StentBoost showed that the stent was divided into 3 sections with 2 fractures (red arrow). C–H, The IVUS images showed that the distal segment of the guidewire was in the false lumen (C), and the stent struts disappeared at 2 places (D, G). IVUS = intravascular ultrasound, LAD = left anterior descending.

A 71-year-old man with hypertension and hyperlipidemia underwent PCI in distal left anterior descending (LAD) artery with a cypher stent measuring 2.5×33 mm (Cordis Corporation, Bridgewater, FL) due to an acute anterior myocardial infarction in October 2006. In September 2012, he was admitted with effort chest pain. The angiogram revealed that the LAD artery had total occlusion, the left circumflex (LCX) artery and RCA had moderate stenosis with good collateral flow to the distal LAD artery. The LAD artery was engaged with a 6-Fr BL3.5 catheter; the HI-TORQUE Pilot 50 guide wire (Abbott Vascular, Santa Clara, CA) and Conquest Pro guide wire (ASAHI INTECC CO,LTD, Vietnam) could not pass the occluded lesion, and then with the support of Finecross MG micro-guide catheter (Terumo Shibuya-ku Tokyo, Japan), the Fielder XT guide wire (ASAHI INTECC CO, LTD, Vietnam) was successfully advanced to the distal part of the total occlusion. A Flextome Cutting Balloon measuring 2.5 × 10 mm was used to dilate the restenosis lesion in the old stent at 6 to 8 atm repeatedly. The angiogram showed that the previous cypher stent in the distal LAD artery was discontinuous (Fig. 2A). StentBoost showed that the stent was divided into 3 sections with 2 fractures (Fig. 2B). The IVUS image showed that stent restenosis was critical and the distal segment of the guide wire was in the false lumen (Fig. 2C) and that the stent struts disappeared at 2 sites (Fig. 2D and G). With IVUS guiding, the guide wire found the true lumen, and a Resolute stent measuring 2.25 × 30 mm was implanted to overlap the 3 mm old distal stent for covering the dissection. The final angiogram showed that the distal flow was thrombolysis in myocardial infarction-3 (TIMI-3) in the LAD artery. The patient was discharged after 3 days, and he was asymptomatic during his 3-year follow-up examination with the optimal medical therapy.

### 2.3 Case 3—stent fracture caused acute myocardial infarction with no typical IVUS image

**Figure 3:**
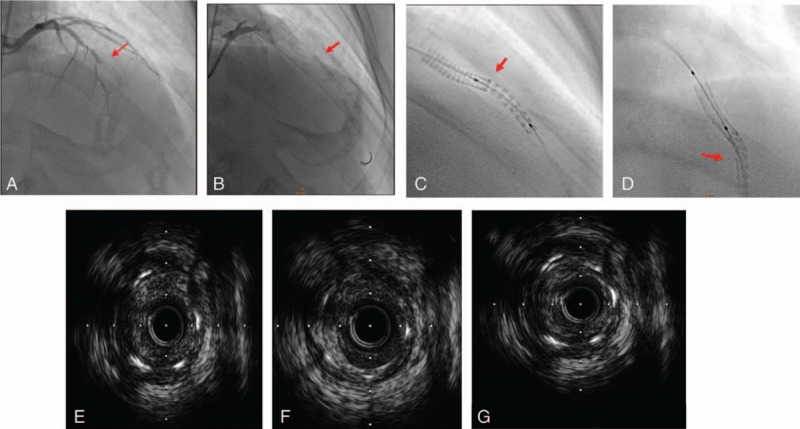
A, Coronary angiography revealed thrombotic occlusion of the stented LAD artery segment (red arrow). B, After thrombus aspiration, coronary angiography showed that the stent struts interrupted close to the overlapping stents (red arrow). C, StentBoost image clearly displayed defected stent struts next to the distal of the 2 overlapped old stents (red arrow). D, Another position showed malalignment of the stent struts (red arrow). E–G, IVUS images showed the stent struts and the decreased number of stent struts from 4 to 5 close to the overlapping of the 2 stents (F). IVUS = intravascular ultrasound, LAD = left anterior descending.

A 65-year-old male with hypertension and a history of smoking had primary PCI to the mid-LAD artery with an Excel stent measuring 3.5 × 18 mm (JWMS, Weihai, China) in August 2006. He had the second PCI to the LAD artery with a cypher stent measuring 3 × 18 mm due to stent restenosis (in segment) in November 2010. On August 15, 2014, he presented with acute anterior wall ST segment elevation myocardial infarction (STEMI) and got admitted to the hospital. The coronary angiography revealed total occlusion in the middle of the LAD artery (Fig. 3A). After successful thrombus aspiration, the stent struts were found to be interrupted close to the overlapping site (Fig. 3B). To confirm this indistinct image, the StentBoost technology was used. The StentBoost image clearly displayed defected stent struts next to the distal of the overlapping site of the 2 stents (Fig. 3C), and another position showed the malalignment of the stent struts (Fig. 3D). IVUS confirmed diffused severe restenosis in the stents and in proximal and distal segments, but the typical stent fracture was not found in IVUS (Fig. 3E–G). In addition, at the place of stent fracture (Fig. 3F), the myocardial bridge was visible. The myocardial bridge may be playing an important role in the stent fracture. Based on the aforementioned results, coronary artery bypass grafting (CABG) was suggested, but the patient refused CABG because it causes damage to a great extent. So, PCI was given to him again. With the StentBoost assistance, 2 adjacent sequential drug eluting stents (DES) Xience V (Abbott Vascular, Santa Clara, CA) measuring 3.0 × 38 mm^2^ and 3.5 × 18 mm^2^ were precisely positioned and overlapped 3 mm to fully cover the lesion. The final angiography demonstrated a successful result with no residual stenosis and TIMI-3 flow. The patient was asymptomatic in 1 year's follow-up examination.

## 3 Discussion

In the bare metal stent era, stent fracture was not a major issue; because of the excessive neointima formation, stent fracture was not found easily. In recent years, with the rapid development of DES and interventional imaging technology, the incidence of stent fracture is increasing and more studies are focusing on stent fractures. The reported incidence of stent fractures varies widely from 0.84% to 18.6% due to many different factors, including the definition of stent fracture, stent type, detection technology, and the rate of angiographic follow-up,^[4–6]^ while it is higher up to 29% at autopsy.^[7]^ According to the previous studies, the contributing factors of stent fracture included technical factors, such as stent overexpansion, stent overlap, stent length, stenting technique, and stent type, and conformability, such as the higher incidence of sirolimus-eluting stents than paclitaxel-eluting stents and everolimus-eluting stents. Anatomic factors (tortuous and angulated vessel) also have a certain effect.^[8]^ Some studies reported that the incidence of stent fracture in stent location varies widely; the most common is the RCA (56.4%), then are the LAD artery (30.4%), LCX artery (10.9%), and saphenous vein grafts (1.7%), while left main stents have almost no fracture.^[1]^

About the relationship between stent fracture and clinical events, stent fractures can cause different clinical presentations including asymptomatic, angina, acute myocardial infarction, and even sudden death. The angiogram showed that it may lead to stent restenosis and also thrombosis requiring target lesion revascularization; some studies also found that stent fracture can lead to coronary artery aneurysms. Stent fractures have been classified into 5 grades: I (single-strut fracture), II (more than 2 struts fractured), III (more than 2 struts fractured with deformation), IV (fracture with transection but without gap), and V (fracture with transection and a gap between stent segments).^[7]^ In the aforementioned 3 cases: case 1 was grade II, the patient was asymptomatic, and an angiography showed mild stent restenosis; case 2 was grade IV, the patient presented with angina, and the angiography showed critical stent restenosis-induced chronic total occlusion; and case 3 was grade V, the patient presented with STEMI, and the angiography showed stent thrombosis. Therefore, the stent fracture might be an important risk factor for stent failure. No consensus is found at present about managing stent fracture. In the 3 cases, case 1 was not intervened, as the patient presented no symptoms; the other 2 cases were given target vessel revascularization. During a mean follow-up of 3.6 years (1.25–6.5 years), the 3 patients had no recurrent angina and myocardial infarction.

In recent years, several stent fracture cases were reported with StentBoost detection.^[9–11]^ StentBoost can improve angiographic visualization of the stent and its relationship with the corresponding vessel lumen by enhancing the x-ray focus of the region where the stent is placed; it has a great advantage in detecting stent morphology. So StentBoost can be used to confirm a stent fracture after high suspicion by an angiographic image. It can identify complete or partial stent fracture. Kim et al^[7]^ reported a case in which StentBoost confirmed the diagnosis of a type IV stent fracture despite the lack of IVUS evidence of stent fracture. Indeed, IVUS may miss diagnosis in some partial fracture cases. In the 3 cases presented in this study, StentBoost clearly showed stent strut fractures with transection and a gap, while IVUS did not find typical stent fractures. The novel fluoroscopic stent visualization techniques have the potential to identify stent strut fractures. The present study was only a single-center, small-sample study; more cases will be needed to confirm the advantages of StentBoost in the future.

Although stent fracture occurs uncommonly, it might be an important risk factor for stent failure. StentBoost can improve angiographic visualization of the stent, which enables the identification of stent fracture, even when stent fracture is sometimes not obvious in IVUS. Compared with IVUS, StentBoost is practical and fast, adds no additional cost, and decreases procedure time.

## References

[1] Mohsen MK Alqahtani A Al Suwaidi J Stent fracture: how frequently is it recognized? Heart Views 2013;14:72–81. 23983912 10.4103/1995-705X.115501 PMC3752880

[2] Kuramitsu S Iwabuchi M Haraguchi T Incidence and clinical impact of stent fracture after everolimus-eluting stent implantation. Circ Cardiovasc Interv 2012;5:663–71. 23011266 10.1161/CIRCINTERVENTIONS.112.969238

[3] Okumura M Ozaki Y Ishii J Restenosis and stent fracture following sirolimus-eluting stent (SES) implantation. Circ J 2007;71:1669–77. 17965483 10.1253/circj.71.1669

[4] Chung WS Park CS Seung KB The incidence and clinical impact of stent strut fractures developed after drug-eluting stent implantation. Int J Cardiol 2008;125:325–31. 17434616 10.1016/j.ijcard.2007.02.033

[5] Lee SH Park JS Shin DG Frequency of stent fracture as a cause of coronary restenosis after sirolimus-eluting stent implantation. Am J Cardiol 2007;100:627–30. 17697818 10.1016/j.amjcard.2007.03.073

[6] Shaikh F Maddikunta R Djelmami-Hani M Stent fracture, an incidental finding or a significant marker of clinical in-stent restenosis? Catheter Cardiovasc Interv 2008;71:614–8. 18360853 10.1002/ccd.21371

[7] Nakazawa G Finn AV Vorpahl M Incidence and predictors of drug-eluting stent fracture in human coronary artery a pathologic analysis. J Am Coll Cardiol 2009;54:1924–31. 19909872 10.1016/j.jacc.2009.05.075

[8] Canan T Lee MS Drug-eluting stent fracture: incidence, contributing factors, and clinical implications. Catheter Cardiovasc Interv 2010;75:237–45. 20025045 10.1002/ccd.22212

[9] Kim MS Eng MH Hudson PA Coronary stent fracture: clinical use of image enhancement. JACC Cardiovasc Imaging 2010;3:446–7. 20394908 10.1016/j.jcmg.2009.06.015

[10] Shinde RS Hardas S Grant PK Stent fracture detected with a novel fluoroscopic stent visualization technique — StentBoost. Can J Cardiol 2009;25:487. 19668785 10.1016/s0828-282x(09)70128-x PMC2732378

[11] Ramegowda RT Chikkaswamy SB Bharatha A Circumferential stent fracture: novel detection and treatment with the use of StentBoost. Tex Heart Inst J 2012;39:431–4. 22719162 PMC3368471

